# An Overview of Physical Exercise and Antioxidant Supplementation Influences on Skeletal Muscle Oxidative Stress

**DOI:** 10.3390/antiox10101528

**Published:** 2021-09-27

**Authors:** Shima Taherkhani, Kosar Valaei, Hamid Arazi, Katsuhiko Suzuki

**Affiliations:** 1 Department of Exercise Physiology, Faculty of Sport Sciences, University of Guilan, Rasht 4199843653, Iran; shimataherkhani@msc.guilan.ac.ir (S.T.); kosar.valaei94@gmail.com (K.V.); 2 Faculty of Sport Sciences, Waseda University, 2-579-15 Mikajima, Tokorozawa 359-1192, Japan; katsu.suzu@waseda.jp

**Keywords:** reactive oxygen species, oxidative stress, skeletal muscle, physical activity, antioxidant supplementation

## Abstract

One of the essential injuries caused by moderate to high-intensity and short-duration physical activities is the overproduction of reactive oxygen species (ROS), damaging various body tissues such as skeletal muscle (SM). However, ROS is easily controlled by antioxidant defense systems during low to moderate intensity and long-term exercises. In stressful situations, antioxidant supplements are recommended to prevent ROS damage. We examined the response of SM to ROS generation during exercise using an antioxidant supplement treatment strategy in this study. The findings of this review research are paradoxical due to variances in antioxidant supplements dose and duration, intensity, length, frequency, types of exercise activities, and, in general, the lack of a regular exercise and nutrition strategy. As such, further research in this area is still being felt.

## 1. Introduction

Free radicals can be defined as any ion, atom, or molecule that can be present independently in the body’s cells of the body. The main feature of these species is an unpaired electron in the outermost part of the shell, which leads to their high reactivity. Since these radicals contain oxygen (O_2_^●−^), they are known as reactive oxygen species (ROS), which include hydroxyl radical (^●^OH), hydrogen peroxide (H_2_O_2_), and superoxide anion (O_2_^●−^) [1,2]. The primary role of O_2_ is in the production of adenosine triphosphate (ATP) through oxidative phosphorylation. However, under stressful conditions such as physical activities compared to normal O_2_ production conditions, due to the inefficiency of mitochondrial complexes I and III, electron transfer from flavin adenine dinucleotide (FADH2) and nicotine adenine dinucleotide (NADH) to electron transfer chain (ETC) is disrupted, which is an influential factor in ROS production, and consequently the production of oxidative stress (OS) under such conditions [3,4]. This would not be valid for all physical activities. According to recent research, regular exercise improves diseases such as dementia, type 2 diabetes, and cardiovascular disease due to its effective action on skeletal muscle (SM). Thus, by performing such activities, the ability of SMs to cope with stress caused by exercise is improved, and this tissue adapts to new conditions [5,6]. However, as ROS expression increase during strenuous activities, the body’s antioxidant capacity declines, causing cell molecules to be destroyed. To deal with these side effects, we usually need to help maintain the function of the body’s endogenous defense systems by consuming nutrients, especially antioxidant supplements [7,8].

This study reviewed the effect of taking antioxidant supplements on ROS produced by physical activity in SM. The first section provides an overview of the generation of free radicals inside the SM and how to deal with them. The significance of physical exercise in the generation of free radicals in SM was addressed in the second section. The third section of this article focused on the influence of antioxidant supplementation on SM. This section was examined from three points of view: adaptations to training, inflammation and muscle damage. The last section also examined disorders related to loss of strength and SM mass (Duchenne muscular dystrophy (DMD) and sarcopenia), antioxidant supplements and physical exercise.

## 2. The Generation of Free Radicals inside SM and Way to Deal with Them

With their daily activities in the body and during the process of oxidative phosphorylation, eukaryotic cells provide the basis for the formation of ROS, especially O_2_^●−^ [9]. During this process, electron carriers produce ATP by transferring electrons from FADH_2_ or NADH to O_2_ [10,11]. This becomes even more important under stressful situations such as physical activities where the SMs are continuously contracted for a long time because the O_2_ consumption of these muscles increases dramatically in such conditions [12]. In essence, the significant source of ROS formation is inefficient electron transport between mitochondrial complexes I and III. In mitochondrial I and III complexes, the primary sites of electron-to-O_2_ leakage are iron-sulfur and semiquinone Q10 clusters. Of course, most of the O_2_ used in the ETC is converted to water (H_2_O) [13]. It has been predicted that the generation of these species rises only two to four times as much as usual in mitochondria during SM contraction. However, during high-intensity exercises, mitochondrial O_2_ demand can rise to 100-fold [14]. When compared to low-intensity, short-duration aerobic activity (i.e., <40 per cent VO_2_max), relatively high-intensity, extended aerobic exercise (i.e., 65–75 per cent VO_2_max) produces more ROS [15]. In general, ROS generated in the mitochondria undergo electron reduction, contributing to around 2 to 5% of the total energy demand by the mitochondria [13,16]. Recent research has demonstrated that during exercise, just 0.15 per cent of the total oxygen used by mitochondria results in the generation of ROS, which is substantially lower than previously thought [17]. After several studies, the researchers concluded that the activity of uncoupling proteins (UCPs), particularly UCP3 in SMs, is the primary cause of the decrease in ROS production in mitochondria compared to the initial estimate of lowering mitochondrial ROS levels [17,18] because these proteins attempt to protect mitochondria from oxidative damage. UCPs seem to be nuclear-encoded transmembrane transporter proteins that are often found in the inner membrane of mitochondria. Although these proteins were discovered in brown adipose tissue for thermogenesis, they are also found in other body tissues, including SM, which may assist regulate the generation of mitochondrial-derived ROS [19]. In addition, although ATP is employed as an energy source for cells, these cells occasionally require thermal energy, and in such cases, UCP typically plays a role in such a way that these proteins prevent mitochondria from forming proton gradient potential. As a result, ATP is transformed straight to heat [20,21]. Of course, mitochondria play an essential role in critical cellular processes such as survival cells and calcium homeostasis regulation. Maintaining the health of this functional organ, particularly in tissues that require much energy for their activities, is critical [22].

The nicotinamide adenine dinucleotide phosphate (NADPH)-oxidase (NOX) mitochondrial enzyme is also involved in the formation of ROS in cells [23]. Approximately five distinct NOX protein homologues have been expressed and detected in various tissues of the body. For example, NOX2 is expressed in SM and is the predominant source of ROS generation in both contractile and resting states [24,25]. Furthermore, this enzyme accelerates the oxidation of the ryanodine receptor (RyR) and the interruption of calcium release by the sarcoplasmic reticulum (SR), resulting in SM contraction failure [26]. In addition to SR, NOX is found in the transverse tubes (TT) of SM. This enzyme’s activity rises in TT in parallel with depolarization, finally resulting in ROS releasing into the SM cytosol and tissue damage [27].

Xanthine oxidase (XO) is another enzyme implicated in the generation of ROS in SM. It has been discovered that the contractile SM cytosol of rats is the location of XO-induced ROS generation. However, since XO levels in human SM are lower than in rats, it is unclear if this enzyme also plays a role in ROS formation. As a result, the need for more research on this subject remains [28].

**Figure 1:**
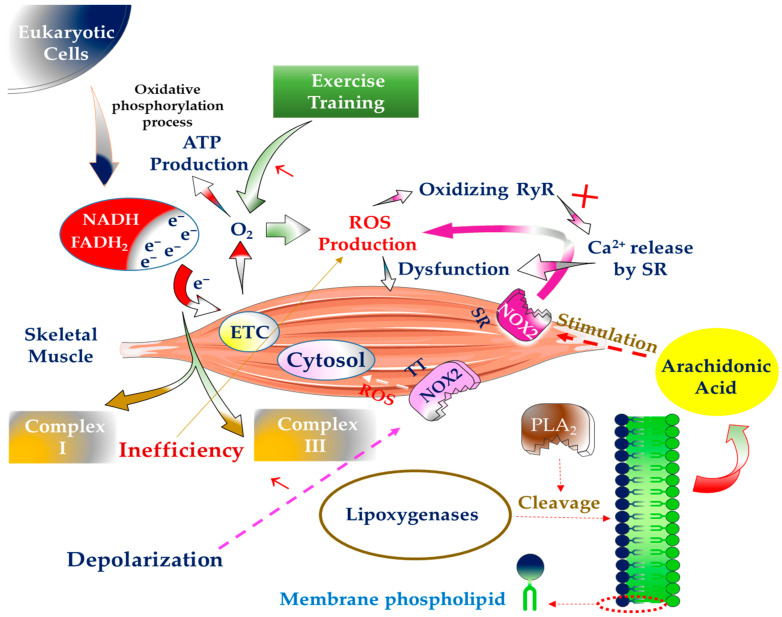
ROS generation sources in skeletal muscle. Reactive oxygen species (ROS), nicotinamide adenine dinucleotide (NADH), flavin adenine dinucleotide (FADH_2_), electron transfer chain (ETC), ryanodine receptor (RyR), sarcoplasmic reticulum (SR), nicotinamide adenine dinucleotide phosphate (NADPH)-oxidase (NOX), transverse tubes (TT), phospholipases A_2_ (PLA_2_).

Other sources, such as arachidonic acid release by phospholipases A_2_ (PLA_2_), have a role in producing ROS in SM. In addition, arachidonic acid is used as a substrate by many ROS-producing enzyme systems, such as lipoxygenase. PLA_2_ cleaves membrane phospholipids, releasing these fatty acids. On the other hand, NOX is activated by PLA_2_ and contributes to increased ROS generation in the cytosol and mitochondria of muscle cells. The produced ROS eventually penetrates the extracellular space and causes damage to this location [29]. It is worth noting that both calcium-independent and calcium-dependent PLA_2_ variants are implicated in ROS generation in SM. The 14 kDa calcium-dependent isoform (sPLA_2_) in the mitochondria is vital for forming intracellular ROS during contractile action. At the same time, the cytosol is the location of ROS generation by calcium-independent enzymes (iPLA_2_) (Figure 1) [30].

The protein myostatin (Mstn), released by myocytes, is another generator of ROS in SM. This protein belongs to the transforming growth factor-β (TGF-β) family, enhancing muscle protein breakdown and impeding muscle cells’ growth path [31]. Nevertheless, this protein is also implicated in the formation of ROS. Therefore, it can make these reactive species through tumour necrosis factor-alpha (TNF-α), canonical Smad3, and nuclear factor kappa B (NF-κB). In addition, activating the ERK mitogen-activated protein kinase (MAPK) and p38 pathways is another mechanism for myostatin to create ROS. However, this occurs in the absence of Smad3 and is predominantly regulated by interleukin-6 (IL-6), TNF-α, XO, and NOX [32].

Nitric oxide (NO), on the other hand, is a main radical produced primary by muscles, whose levels rise rapidly during contractile circumstances. It is typically obtained by three separate isoforms of NO synthase (NOS1, NOS2, and NOS3) and from the amino acid L-arginine, which usually is expressed by two neuronal NOS (nNOS/NOS1) and endothelial NOS (eNOS/NOS3) isoforms [33]. Fast-twitch muscle fibres, typically seen in the muscular sarcolemma, express nNOS, whereas eNOS is found in muscle mitochondria. However, in inflammatory situations, the inducible NOS isoform (iNOS/NOS2) is also likely expressed in skeletal muscle fibres. This radical can interact with O_2_^−^ to generate peroxynitrite, a potent oxidizing agent that can gradually deplete the thiol groups in the cells. The cellular redox signalling pathways alter concurrently with this event [30]. In actuality, with the help of NADPH and iron and 5,6,7,8-tetrahydrobiopterin cofactors, these NOSs may convert L-arginine to L-citrulline and NO [34].

Following the formation of O_2_^●−^ in various regions of SMs such as mitochondria, SR, cytosol, and TT, this radical spontaneously or via superoxide dismutase (SOD) enzyme leads to the creation of H_2_O_2_. As H_2_O_2_ is a mild oxidant with a long half-life, it may pass cell membranes and cause harm to a variety of cells throughout the body [28]. In addition, breaking the balance between cells’ ability to neutralize ROS and overproduction of these species causes OS in various tissues. Nevertheless, if the concentration of ROS within the cells is maintained at a reasonable level, it can help cells to proliferate, adapt, grow, and differentiate. Maintaining proper ROS levels is the consequence of cells eliminating ROS through the actions of antioxidant enzymes such as catalase (CAT), SOD, peroxiredoxin (PRX), and glutathione peroxidase (GPX). Of course, limiting ROS-generating electron leakage is another strategy to protect cells from oxidative damage [11,35]. In addition to enzymatic antioxidants (including SOD, GPX, CAT, peroxiredoxins (PRXs), glutaredoxins (GRX), and thioredoxin (TRX) reductases, glutathione reductase (GSR)), non-enzymatic antioxidants or low-molecular-weight antioxidants (LMWAs) are also responsible for regulating ROS in the body [36]. LMWA can be divided into two general categories: fat-soluble and water-soluble; which are mainly synthesized endogenously in the body (such as uric acid, coenzyme Q (CoQ10), lipoic acid, bilirubin, dipeptide carnosine (β-alanyl-histidine), estrogen and melatonin), or through the consumption of diet and nutrients (such as vitamins C, E, carotenoids, phenolic compounds) enter the body. Of course, it is important to note that the antioxidant effects of these compounds have not been fully established in vivo, and it is even possible that several of them in combination may induce antioxidant effects. Hence, countless studies must be done to reach a definite conclusion [37].

## 3. The Significance of Physical Exercise in the Generation of Free Radicals in SM

In 1954, scientists identified the existence of ROS in live cells. However, it was discovered in the late 1970s that there was a correlation between ROS and physical activity [38]. Davies et al. reported in 1982 that muscular activity increases the formation of ROS and free radicals in contracting SM [39]. Kondo et al. (1991) reported that commencing exercise after extended rest might likewise enhance ROS in SM fibres [40]. In reality, this viewpoint dates back over 40 years, when researchers found OS in both blood and SM due to high-intensity anaerobic, long-term endurance, eccentric, and resistance activities [15,41]. Early investigations showed that SM generates OS during and after exercise due to ROS production in this tissue. However, this is likely to alter with time. Later, the researchers discovered that ROS generated during contractile processes did not damage SM function and played an essential role in controlling the signals required for adaptive responses to exercise. As a result, subsequent research has concentrated on redox signalling in various sporting conditions [42]. One of the complications of SM contraction due to overproduction of ROS is muscle atrophy. When ROS levels in SM rise, FoxO and NF-KB activity is increased in muscle cells. Following this, muscle really interesting new gene (RING)-finger protein 1 (MuRF-1) and muscle atrophy F-box (MAFbx) are activated [43,44]. By activating these two, various SM proteins, including myosin-binding protein C, titin, myosin heavy chain, troponin, myosin light chains 1 and 2, and nebulin, are broken down, leading to muscle atrophy [45]. Regular physical activity has also improved CVD, psychiatric, musculoskeletal disorders, neurological and pulmonary diseases [46]. The intensity and duration of physical exercises on either side are critical in this respect. It has been demonstrated that increasing these two factors significantly go up the possibility of enhanced ROS production during SM contraction and damage to various cellular components of the body [47], which leads to disturbances of muscle redox balance, muscle fatigue, OS, and impaired athletic performance [48]. One study reported that regular physical exercises with temporary ROS production are important in adapting to exercise-induced stress and optimal physiological function. However, sports capacity decreases mainly under stressful conditions (overtraining, physical inactivity, and excessive muscle-damaging exercise) [49]. On the other hand, muscle fatigue usually occurs after intense and repetitive exercise, which leads to impaired SM function. Under such conditions, Ca^2+^ release from the SR and the maximal production force of the contractile apparatus diminish due to increased concentrations of free magnesium (Mg^2+^) and inorganic phosphate and decreasing ATP, glycogen and pH levels [50].

## 4. The Influence of Antioxidant Supplementation on SM

### 4.1. In Terms of Adaptations to Training

The efficacy of using antioxidant supplements for training adaptation in SM has been questioned in recent years. Some research has suggested that these substances might help with post-exercise recovery. However, some research suggests that these supplements may not only enhance post-workout adaptations but may slow them down [51]. Moreover, both experienced and untrained athletes’ redox homeostasis frequently changes due to anaerobic and aerobic activities. It is not easy to pinpoint the processes that cause OS during diverse sports activities. Several studies, however, have demonstrated that because SM makes up the majority of the body’s muscles, this muscle may be recognized as ROS-affected tissue. Of course, ROS is produced by SM tissue, and other tissues in the body such as the lungs, heart, and liver can produce ROS during exercise [49]. On the other hand, researchers in various studies have shown that cooperation between antioxidant supplements and endogenous antioxidants leads to the elimination or neutralization of ROS by increasing the ability of SM fibers. Therefore, maintaining antioxidant levels is very important to prevent muscle breakdown [48].

Paulsen et al. invited strength-trained recreational men and women for their study. For ten weeks, these participants were given antioxidant supplements of vitamins E and C (235 and 1000 mg per day, respectively). They also performed heavy-load resistance training (RT) four times a week. No increase in muscle mass of these subjects was observed after taking antioxidant supplements [52]. Additionally, one research found that consuming large dosages of vitamin C (500 mg kg^−1^) for 14 days inhibited hypertrophy of overworked muscles in Wistar rats. This event is accompanied by a reduction in the ROS-regulated p70S6K and ERK1/2 (regulators of skeletal muscle hypertrophy) [53]. Theodorou et al. employed males who exercised recreationally as volunteers in another research. These men were given vitamins E and C (400 IU and 1000 mg per day, respectively) for 11 weeks. They, on the other hand, conducted eccentric exercise for four weeks and twice a week. Finally, it was demonstrated that these supplements had no impact on muscular function or recovery following exercise [54]. Dutra et al. employed healthy and non-smoking women as subjects to evaluate muscle thickness and function after ten weeks of twice-weekly strength training (ST). They consumed vitamins E and C (400 IU and 1000 mg daily, respectively) during the ST period. Peak torque and total work were assessed in this study and were lowered due to taking antioxidant supplements. However, over the ST period, these variables improved [55].

Besides that, it has been well established that CoQ10 levels can be increased in tissues such as the heart, SM, kidneys, and liver of high-intensity active people by taking CoQ10 supplements and developing oxidative phosphorylation process helps to improve performance. In this regard, review research found that CoQ10 supplementation can alter athletes’ aerobic performance. However, the findings of the trials are paradoxical since taking this supplement alone or in conjunction with vitamins E and C failed to increase SM metabolism in multiple placebo-controlled experiments [56]. Cooke et al. likewise recruited both trained and untrained men and women as participants. They were given 200 mg of C_O_Q10 every day for 14 days. WT GXT and isokinetic tests were utilized in this investigation. They determined that higher CoQ10 levels did not affect the individuals’ physical endurance [57].

One study looked into the relationship between consuming antioxidant supplements and boosting VO_2_max via the influence on SM mitochondria. Taub et al. recruited healthy and sedentary adults for their investigation. For three months, these participants were fed 20 g of dark chocolate. They also went for a stationary bike ride. Finally, the researchers concluded that improving mitochondrial function might enhance these people’s VO_2_max [58].

Biochemical and structural adaptations to ST can help improve health and physical function by increasing SM mass and strength. Biochemical adaptations are more closely linked to effective post-exercise stress management systems such as endogenous antioxidants and heat shock proteins (HSPs) [51,59]. In stressful circumstances like inflammation, OS, ischemia, hypoxia, physical exercise, and fever, HSPs can protect the body’s cells by possibly degrading damaged proteins, initiating protein folding, repairing, and refolding misfolded peptides. Indeed, the expression of these proteins rises with increased ROS generation, which can safeguard the body’s cells owing to their roles in the destruction of irreversible peptides, transport of organelles across membranes, polypeptide folding, and assembly [60]. Moreover, following high-intensity ST, HSPs accumulate in damaged regions of SM, leading to a decrease in productive capacity, which can be regarded as a helpful element in restoring muscular function and promoting recovery [51]. Furthermore, it has been demonstrated that after 5–11 weeks of ST, the muscles of the upper and lower limbs undergo an increase in HSPs [61].

Bowtell et al. looked at trained men to see how their SM strength changed following laborious exercise. These patients did 10 sets of 10 repetitions with a single knee extension at 80% of their maximal repetition. They also drank 30 mL (twice daily) of Montmorency cherry juice for one week before and 48 h after exercise. The results revealed that this antioxidant supplement improved muscular isometric strength following intensive exercise [62]. McLeay et al. investigated exercise-induced muscle damage (EIMD) in healthy women in another investigation. The subjects were given a blueberry smoothie 5 and 10 h before EIMD and immediately, 12 and 36 h afterwards. According to the researchers, the smoothie increased isometric muscular strength recovery due to lower ROS generation, according to the researchers [63]. Furlong et al. investigated SM strength adaptations in untrained people in another investigation. This study participant participated in a RT regimen (3 times per week, two sets of 10 repetitions per movement, for 12 weeks). This research examined 1-RM leg and chest press, isokinetic strength (knee extension and flexion, elbow), balance, and perception of recovery. During the RT, subjects were also given antioxidant supplements, a proprietary herbal/botanical combination (1575 mg, 2 times a day), and Aphanizomenon flos-aquae extract (1000 mg, 3 times daily). Finally, the researchers discovered no variations in the patients’ balance, strength, or muscular function [64].

### 4.2. In Terms of Inflammation

SM is one of the most critical tissues in the body, and it is particularly vulnerable to inflammation and OS during strenuous activity. Under these conditions, muscle mitochondria are often exposed to contusions resulting from casual/intentioned contacts, mechanical stress from repetitive contractile activity, and intensified respiratory function [65]. Then, as a defense mechanism against OS, infections, and inflammation, neutrophils are recruited to restore SM tissue. Initially, substances produced from injured muscle, such as myoglobin and creatine kinase (CK), cause neutrophils to be mobilized in SM. Next, neutrophils and macrophages produce inflammatory cytokines delivered to the muscle’s damaged region [66]. Neutrophil activation is dependent on the formation of NOX via ROS generation and the fusing of granules into the phagosomal membrane. NOX is efficient in increasing ROS generation during a respiratory burst, particularly O_2_^●−^ [42]. As a consequence, neutrophil phagocyte cell debris, which is commonly responsible for respiratory bursts. H_2_O_2_ is then formed as a result of O_2_^●−^ dismutation by the enzyme SOD. Finally, H_2_O_2_ reacts with the chloride to form chloramine and hypochlorous acid (HOCl) via the action of myeloperoxidase (MPO) [67]. As potent oxidants, HOCl and ROS play a significant role in the phagocytosis of cell debris from injured muscle [68]. Furthermore, since neutrophils successfully secrete inflammatory cytokines from the afflicted region, including TNF-α, IL-6, and IL-8, they activate macrophages and increase phagocytic activity, as extracellular messengers play an essential role. SM tissue is repaired as a result of this process [69]. Carrera-Quintanar et al. chose university-level athletes as subjects for a research study. For the first 21 days, the athletes drank an almond beverage enriched with vitamins C and E and *Lippia citriodora* antioxidant extract. They were then put through a 2000-m running test. The researchers concluded that antioxidant beverages might protect neutrophils from oxidative injury to SM during exercise [70].

### 4.3. In Terms of Muscle Damage

ROS are primarily created in damaged muscles. In reality, when muscle tissue is destroyed, the redox state of the muscle is disturbed. In such cases, the involvement of non-muscular ROS sources such as phagocytic white blood cells is emphasized. Neutrophils and macrophages have been shown to assault SM after injuring muscle fibers [71,72]. Although this promotes muscle fiber regeneration, it also causes phagocytic cells to produce ROS. This is not in the best interests of SM since even unharmed muscle cells are injured during this process, resulting in an additional injury to the muscle fibres [73]. The degree of muscle damage induced by OS is generally proportional to the quantity of ROS present. While high concentrations of these species cause muscle injury, low to moderate concentrations cause muscle remodulation and signalling pathway activation [74].

Furthermore, it has been proven that regular exercise with the appropriate intensity and duration has numerous benefits in human life, but if the intensity of these activities increases, these effects may be reversed. This is especially true for athletes because, during strenuous physical exercise, the SM of these people is usually one of the most important tissues at risk of injury. This is also true for athletes who have been sedentary for an extended period and have recently begun training [75]. Muscle damage in these situations is mainly caused by an accumulation of free radicals and OS, producing delayed onset muscle soreness (DOMS) in this muscle. Thus, DOMS refers to the soreness commonly felt after engaging in unusual and severe athletic activities. Various research by exercise physiologists has shown that DOMS lasts typically 5 to 7 days, while the accompanying pain begins 12 to 24 h after activity and peaks within three days [76] In review research, Antonioni et al., in a review study, noted that athletes’ performance could be affected by exposure to severe stress caused by high-level exercise. Increased ROS production in their SMs is the cause. However, endogenous antioxidants help with the upkeep of redox homeostasis and do not affect athletes’ performance and do not cause muscle injury if ROS levels are kept at a particular threshold [49].

The effect of vitamin E supplementation on OS and muscle damage caused by physical exercise is unclear. Despite its positive effect on lipid peroxidation, some studies have shown that this supplement does not improve muscle damage in endurance athletes. On the other hand, other studies have proven the effectiveness of this supplement in improving muscle damage caused by exercise. It is speculated that different doses of supplementation and the lack of a standardized protocol are the reasons for these conflicting results [77]. In a meta-analysis study published by Stepanyan et al., it was found that exercise-induced muscle damage did not improve with vitamin E intake [78]. Rakitzi et al. used running athletes as subjects in their research. They took 200 mg of vitamin C and 600 mg of vitamin E daily for 5 weeks. The researchers concluded that muscle damage was reduced in these athletes [79]. For 12 weeks, Zoppi et al. studied soccer players. Every day, they were given 1000 mg of vitamin C and 800 mg of vitamin E. These athletes’ muscle damage and OS were minimized. Simultaneously, their athletic performance remained unchanged [80]. On the other hand, Dawson et al. investigated several antioxidant supplements in well-trained runners and had disparate outcomes. They administered 500 to 1000 mg of vitamin C and 750 to 1500 mg of vitamin E per day to their volunteers. However, they found no effect on muscle damage or OS in these athletes [81].

Nieman et al. assessed muscle damage and physical function in well-trained runners using a quercetin antioxidant supplement. For three weeks, the individuals took the supplement at a dose of 500 mg each day. Finally, this antioxidant was discovered not to affect physical function or muscle damage [82]. Kon et al. offered kendo athletes a CoQ10 antioxidant supplement (300 mg/day) for 20 days. As a result, despite no change in OS, the rate of muscle damage was reduced [83]. Another research looked at CoQ10 to see how it affected rugby participants’ physical function, OS, and muscle damage. For one month, the individuals were given this supplement at a dosage of 200 mg per day. Finally, it was discovered that this supplement had no influence on the variables investigated [84]. In their study, O’Fallen et al. utilized young men and women as participants. They exercised eccentrically and were given the antioxidant supplement quercetin (100 mg daily). The researchers discovered no changes in the patients’ muscle damage or inflammatory indicators (arm edema, strength loss, elevated CK, and muscle discomfort) [85].

## 5. Disorders Related to Loss of Strength and SM Mass, Antioxidant Supplements and Physical Exercise

On either side, several disorders, such as DMD and sarcopenia, are associated with decreasing strength and SM mass.

### 5.1. DMD, Antioxidant Supplementation, and Exercise

DMD is a deadly, progressive, and recurring X-linked illness. The primary cause of this condition is a deficiency in the dystrophin protein. This protein works as a powerful stabilizer of muscle fibers during muscular contraction, and it is vital in linking cytoskeleton muscle fibers to the extracellular matrix. This condition causes inflammation, Injuries due to SM contraction, OS, and mechanical instability [86]. In DMD, mechanical muscle instability is strongly related to myofiber reconstruction and destruction and activation of muscle stem cells or satellite cells. Satellite cells can begin to differentiate and proliferate in phases by activating when skeletal muscle is damaged. These cells subsequently generate new regeneration fibers (along with nuclei in the centre), and the reservoir of satellite cells gradually empties, increasing OS and inflammation [87].

DMD can be influenced by nutritional and exercise therapy strategies. Still, researchers have not yet provided a definitive answer on the effectiveness of treatments for this muscle disease despite much research in this area.

ROS can affect muscle force production through oxidation contractile and excitation-contraction (E-C) coupling proteins, leading to muscle fatigue. However, studies have shown that antioxidant supplements can reduce the negative impact of overproduction of these reactive species to some extent [88]. In this regard, various researchers began to study the effect of antioxidant supplements on mdx mice by conducting clinical trials [89]. It was shown that SM fatigue resistance in these mice was improved by overexpression of the antioxidant CAT enzyme [90]. Overall, the initial results of this research were disappointing and even negative [89]. In one study, researchers investigated antioxidant supplementation of green tea extract (GTE) on mdx mice (aimed at counteracting OS in dystrophic muscle cells). Finally, it was shown that this supplement could reduce necrosis and improve function in muscle tissue [91]. On the other hand, the antioxidant supplements melatonin, NAC and CoQ10 have also been considered by researchers because of their potential ability to control ROS overproduction in dystrophic muscles. Although studies indicate a positive effect on DMD improvement, definitive results are not yet available [92,93,94].

On the other hand, several studies have examined the effect of exercise on DMD. In one of these studies, four DMD patients with the least antigravity strength quadriceps trained one quadriceps submaximally four or five days per week for six months. In this exercise, the Cybex isokinetic trainer was used, and the subjects had to stretch their knees completely from 90 degrees. Of course, it is noteworthy that only one knee was trained, and the other knee was inactive. This study showed that although it has a limited effect on improving DMD, this exercise has no adverse effect on it [95].

### 5.2. Sarcopenia, Antioxidant Supplementation, and Exercise

Another disorder associated with decrease SM strength, mass, and endurance is sarcopenia, which develops with age. The disorder is mainly connected with physical impairment and, in extreme cases, death [96]. Under these conditions, SM contraction is disrupted, resulting in an imbalance in the synthesis and breakdown of SM protein. On the other hand, chronic inflammation and OS play an essential role in developing apoptosis, poor muscle protein metabolism, and mitochondrial dysfunction. According to the European Working Group on Sarcopenia in Older People (EWGSOP), there are three skeletal, muscular weakening levels in patients with sarcopenia: pre-sarcopenia, sarcopenia, and severe sarcopenia [97]. Thus, although physical function and muscle strength do not decrease in the pre-sarcopenia phase, muscle mass is lost to a minimal extent. Although sarcopenia in the elderly cannot be avoided, it can be mitigated to some extent by various treatment measures such as diet and exercise [98]. Regular exercise is an essential strategy to avoid sarcopenia, but many older individuals are unwilling to exercise due to sarcopenia’s complicated nature, with its disease-related and age-related signs [99]. Liang et al. (2021) studied male Sprague-Dawley rats. Lipopolysaccharide (LPS) muscle injury was used to produce ROS and sarcopenia. They were tested on the treadmill and their grip strength, and the antioxidant supplement employed in this study was Bletilla striata (BSP). Finally, the authors observed that BSP improved grip strength, muscular mass, and muscular endurance in these rats (Table 1) [100].

One of the dangers of free radicals on body tissues, especially SM, is decreased muscle mass and size with age. Various muscle fibres, including type 1 fibres (role in oxidative metabolism) and type 2 fibres (important in the glycolytic process), make up SM. Among these, type 2 fibres are mainly associated with decreased muscle mass during the sarcopenia process. In fact, with age, type II muscle fibres usually convert to type I fibres. During this conversion, myosin heavy chain protein synthesis and muscle strength are reduced [101,102]. With this reduction, the ability to contract muscles decreases that even a person can hardly perform their daily activities. Researchers attribute these events to an increase in age-related free radicals, which significantly reduces the body’s endogenous antioxidant ability to fight these radicals [103]. Therefore, it is recommended that it is possible to help maintain SM mass by consuming nutrients, mainly antioxidant supplements. These supplements can combat complications of aging, including sarcopenia, by counteracting anabolic muscle resistance and reducing OS indices (in both animals and humans) [101]. The results of studies are contradictory in the effectiveness of antioxidant supplements on SM function. Studies have shown that vitamin C can help boost SM antioxidant defenses and improve sarcopenic performance (grip strength, DXA-measured fat-free mass, grip strength, and explosive leg power), which usually affects the activity of SOD and CAT enzymes [104]. One study even showed that this vitamin could reduce exercise-induced muscle damage [105]. Researchers obtained this result when researchers examined the antioxidant vitamins C and E [106]. Another study showed that taking a combination of antioxidant supplements such as vitamin A, selenium, and zinc stimulated the anabolic response of old muscles to leucine and subsequently inhibited protein breakdown in rats [107]. Some studies have pointed to the opposite effect of antioxidant supplements on skeletal muscle. In this regard, in one of these studies, it was shown that mitochondrial antioxidant enzymes were reduced using a 14-week intake of α-lipoic acid and vitamin E [108]. In another study, researchers concluded that SM function was not affected by the antioxidant supplementation of tocopherol and ascorbic acid and that exercise-induced muscle damage was not improved by taking these vitamins [99].

There is evidence that exercise plays a vital role in the body, improving general health, endurance, immune function, muscle mass and strength with age [109]. One of the most critical and influential types of exercise on SM is aerobic exercise. In this regard, researchers have shown that mitochondrial biogenesis and fission 1 protein (FIS1) can be increased after 12 weeks of aerobic exercise [110,111]. In addition to aerobic exercise, studies have examined the role of resistance exercises on SM. After comparing these two types of training (aerobic and resistance), a study concluded that the SM mass and strength probably respond more to resistance training than aerobic training. Consequences such as low participation of subjects, increased risk of injury and even fatigue in performing resistance training are more common [112]. Defects in the mTOR signaling pathway (important in SM protein synthesis) are also likely to be seen in the elderly after resistance training [113]. Therefore, since neither aerobic exercise nor resistance training can positively affect age-related sarcopenia alone, it is better to prescribe a combination of both types of exercise for the elderly. In this way, doing circuit exercises is the best type [114]. In this regard, a study showed that 12 weeks of circuit exercise could improve balance and gait functions and help improve isokinetic muscle functions [112]. One study found that the strength and muscle mass of subjects between the ages of 20 and 74 who did cycle exercise improved [115]. On the other hand, researchers examined the combined effect of taking the antioxidant supplement resveratrol and 12 weeks of exercise in people aged 65–80 years. These researchers finally showed that the combination of diet and exercise strategies has a more significant anabolic role on SM in these individuals than exercise alone [116].

**Table 1:** The influence of antioxidant supplementation on skeletal muscle (adaptations to training, inflammation, muscle damage).

Reference	Subjects	Antioxidant Supplementation	Exercise Training	Results
Paulsen et al. [55]	Strength-trained recreational men and women	Vitamins E and C (235 mg per day and 1000 mg per day, respectively) for ten weeks	Heavy-duty resistance training four times a week	No increase in muscle mass
Makanae et al. [56]	Wistar rats	Vitamin C (500 mg kg^−1^) for 14 days	Mechanical overload	Decrease of hypertrophy of overworked muscles
Theodorou et al. [57]	Exercised-recreational men	Vitamins E and C (400 IU and 1000 mg per day) for 11 weeks	Eccentric exercise for four weeks and twice a week	No impact on muscular function or recovery following exercise
Dutra et al. [58]	Healthy and non-smoking women	Vitamins E and C (400 IU and 1000 mg daily, respectively)	Ten weeks of twice-weekly strength training (ST)	Decrease of peak torque and total work due to taking antioxidant supplements
Cooke et al. [60]	Trained and untrained men and women	200 mg of COQ10 every day for 14 days	WT GXT and Isokinetic tests	Higher CoQ10 levels did not affect the individuals physical endurance
Taub et al. [61]	Healthy and sedentary adults	20 g of dark chocolate for three months	Stationary bike ride	Increase of VO_2_max
Bowtell et al. [65]	Trained men	30 mL (twice daily) of Montmorency cherry juice for one week before and 48 h after exercise	Ten sets of 10 repetitions with a single knee extension at 80% of their maximal repetition	Improvement of muscular isometric strength following intensive exercise
McLeay et al. [66]	Healthy women	Blueberry smoothie 5 and 10 h before EIMD and immediately, 12 and 36 h afterwards	Exercise-induced muscle damage (EIMD)	Increase of isometric muscular strength recovery
Furlong et al. [67]	Untrained people	Proprietary herbal/botanical combination (1575 mg 2 times a day), and Aphanizomenon flos-aquae extract (1000 mg 3 times daily)	Resistance training regimen (3 times a week, two sets of 10 repetitions per movement, for 12 weeks)	No variations in the patients balance, strength, or muscular function
Carrera-Quintanar et al. [73]	University-level athletes	Vitamins C and E and Lippia citriodora antioxidant extract	2000-m running test.	Protect neutrophils from oxidative injury to skeletal muscle
Rokitzki et al. [82]	Running athletes	200 mg of vitamin C and 600 mg of vitamin E daily for five weeks	-------------------	Decrease of muscular damage
Zoppi et al. [83]	Soccer players	1000 mg of vitamin C and 800 mg of vitamin E for twelve weeks	-------------------	- Decrease of muscle damage and OS - No improvement in athletic performance
Dawson et al. [84]	Well-trained runners	500 to 1000 mg of vitamin C and 750 to 1500 mg of vitamin E per day	-------------------	- No improvement on muscle damage or OS
Nieman et al. [85]	Well-trained runners	Quercetin antioxidant supplement (500 mg/day) for three weeks	-------------------	- No improvement in physical function or muscle damage
Kon et al. [86]	Kendo athletes	CoQ10 (300 mg/day) for 20 days	-------------------	- No improvement in OS concentration - Decrease of muscle damage
Orlando et al. [87]	Rugby participants	CoQ10 (200 mg/day) for 1 month	-------------------	- No improvement in physical function, OS, and muscle damage
O’Fallen et al. [88]	Young men and women	Quercetin (100 mg daily)	-------------------	- No improvement in muscle damage or inflammatory indicators (arm edema, strength loss, elevated CK, and muscle discomfort)
Liang et al. [103]	Male Sprague-Dawley rats	BSP supplementation	Treadmill and grip strength tests	Improvement of grip strength, muscular mass, and muscular endurance

## 6. Conclusions

It is commonly understood that skeletal muscle oxygen requirements rise dramatically during exercise. Because these muscles are used the most in sports, they constantly contract and absorb more O_2_. Of course, the intensity and duration of exercise are essential and influential factors in this increase. Therefore, performing moderate to high-intensity exercise quickly compared to low to moderate intensity and long skeletal muscle oxygen needs will be greater. One of the most severe repercussions of excessive exercise is the overproduction of ROS, which can harm many tissues in the body, including skeletal muscle. Some of these lesions include sarcopenia, and DMD. One strategy to avoid this damage is to take antioxidant supplements and exercise in varying amounts and durations, as discussed briefly in this article. In general, although many studies have looked at antioxidant supplements (vitamins C, E, resveratrol, and CoQ10) and exercise (aerobic, resistance, or concurrent exercise), the results are contradictory. Hence, achieving a standard protocol is still out of reach, and we strongly recommend that researchers address this issue further in the future. They should consider a wide range of antioxidant supplements with different doses and duration and sports activities with different intensities and duration.
